# Insights into photovoltaic properties of ternary organic solar cells from phase diagrams

**DOI:** 10.1080/14686996.2018.1509275

**Published:** 2018-09-25

**Authors:** Mohammed Makha, Philippe Schwaller, Karen Strassel, Surendra B. Anantharaman, Frank Nüesch, Roland Hany, Jakob Heier

**Affiliations:** a Laboratory for Functional Polymers, Empa, Swiss Federal Institute for Materials Science and Technology, Dübendorf, Switzerland; b Institut des Matériaux, Ecole Polytechnique Fédérale de Lausanne EPFL, Lausanne, Switzerland; c Institute of Chemical Sciences and Engineering, Ecole Polytechnique Fédérale de Lausanne EPFL, Lausanne, Switzerland

**Keywords:** Ternary organic solar cells, phase diagram, cyanine dye, 50 Energy Materials, 102 Porous / Nanoporous / Nanostructured materials, 206 Energy conversion / transport / storage / recovery, 306 Thin film / Coatings

## Abstract

The efficiency of ternary organic solar cells relies on the spontaneous establishment of a nanostructured network of donor and acceptor phases during film formation. A fundamental understanding of phase composition and arrangement and correlations to photovoltaic device parameters is of utmost relevance for both science and technology. We demonstrate a general approach to understanding solar cell behavior from simple thermodynamic principles. For two ternary blend systems we construct and model phase diagrams. Details of EQE and solar cell parameters can be understood from the phase behavior. Our blend system is composed of PC_70_BM, PBDTTT-C and a near-infrared absorbing cyanine dye. Cyanine dyes are accompanied by counterions, which, in a first approximation, do not change the photophysical properties of the dye, but strongly influence the morphology of the ternary blend. We argue that counterion dissociation is responsible for different mixing behavior. For the dye with a hexafluorophosphate counterion a hierarchical morphology develops, the dye phase separates on a large scale from PC_70_BM and cannot contribute to photocurrent. Differently, a cyanine dye with a TRISPHAT counterion shows partial miscibility with PC_70_BM. A large two-phase region dictated by the PC_70_BM: PBDTTT-C mixture is present and the dye greatly contributes to the short-circuit current.

## Graphical Abstract

**Figure:**
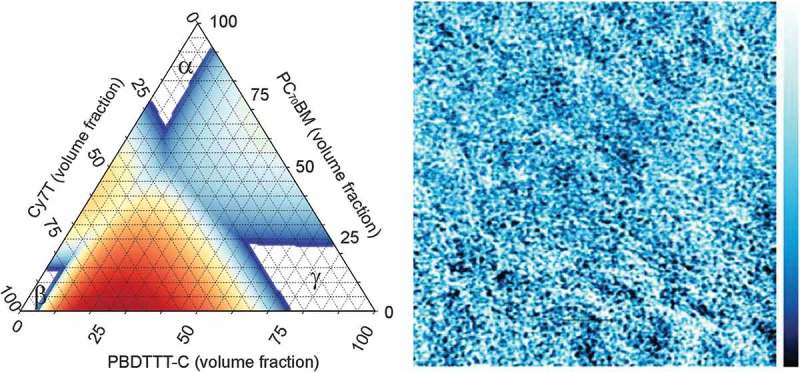

## 1. Introduction

Organic solar cells (OSCs) are attracting great attention thanks to their advantages of low-cost, flexibility and light weight [1,2]. The most frequently used device film morphology for OSCs is the bulk heterojunction (BHJ), consisting of donor- and acceptor rich phases separated on the nanoscale. Design and synthesis of new organic semiconductors, optimization of the device architecture and a deeper understanding of the physical processes increased the certified power conversion efficiencies beyond 11% [3–5].

However, the most commonly used conjugated polymers have limited absorption bandwidths and the fullerene derivatives, frequently used as electron acceptors, show weak absorption in the visible. Therefore, the narrow overlap between the absorption band of a single BHJ device with the solar spectrum results in a restrained device performance. To overcome this absorption limitation, several strategies were examined. Designing new low-bandgap (co)-polymers or combining materials with different bandgaps in a tandem cell are two possibilities to harvest more photons [6,7]. Another promising approach to increase performance is adding a third component into a binary system, resulting in a ternary blend. In contrast to tandem solar cells, ternary blend solar cells keep the simple device architecture and easy processing of a binary blend solar cell while broadening the absorption range. The third component polymers or small molecules can be used either as donor or acceptor [8–14]. Depending on its properties, the third component can contribute to a better performance in different ways. Enhancing the short circuit current density (J_sc_) is possible by adding a component with a complementary absorption profile [15–19]. In addition, several studies showed that also the open circuit voltage (V_oc_) can be tuned by the third component [20–22].

Following the recent literature, the most efficient ternary solar cells today are based on non-fullerene acceptors, with reported efficiencies exceeding 13%. The working principle here is based on two compatible acceptors [23], two compatible donors [24], or cascading energy levels [25]. In another approach, a third liquid crystalline component was added to serve as a morphology regulator [26].

So far, only few investigations have addressed the correlation of the underlying photophysical processes in ternary blend OSCs to phase morphology [20–22]. Already in binary blend systems, a highly optimized morphology is necessary to efficiently generate and extract charge carriers. Ternary blends can form an even larger variety of morphologies, depending on the ratio and the interactions between the components [2,17]. The complexity of the system is substantially increased. The efficiency of electronic processes such as exciton dissociation, charge transfer, energy transfer or charge transport depends on the arrangement of the individual phases and on the relative energetic position of the sensitizer’s electronic levels. Processes specific to ternary blends are described by cascade charge transfer [27], parallel-linkage or by the alloy model [28,29]. Each of them requires an appropriate morphology [2]. In the binary blend, charge transport occurs through interconnected channels of the acceptor and donor phase. Several studies have shown that adding a third component up to a certain amount leads to an increase in the efficiency, followed by a drop at higher loadings. This drop is often reasoned to be due to a change in the morphology [10,17,30]. This dependence makes it necessary that electro-optical properties and morphology are synchronized and it can be hypothesized that controlling the morphology is the key to obtain high performance ternary blend solar cells [30–35].

Finding the ideal three-component composition by trial and error is a cumbersome process. Phase diagrams are a fundamental tool to better understand and control the morphology of multi-component mixtures and are common in polymer science. Phase diagrams also give valuable insights into the composition of a phase (solubility of minority components in the majority component) [36]. However, they have been employed only recently to explain the morphology of a BHJ-blend in a solar cell device [37]. A couple of reasons can be made accountable for this. First of all, BHJs are typically fabricated by solution casting, where the final morphology is the result of a fast solvent quench that can hardly follow thermodynamic equilibrium. Strictly spoken, a system with three solid components and a common solvent is punctiliously described by a quaternary phase diagram. During solvent evaporation the composition and consequently the thermodynamic driving forces change continuously and most frequently a particular morphology freezes in before thermodynamic equilibrium is reached as represented by a phase diagram. Still, the ternary phase diagram can be a good approximation when looking at the final film. Second, different competing mechanisms lead to the structure formation in BHJs. Crystalline materials may form nanostructured morphologies when crossing the liquidus [38]. A competing mechanism is spinodal decomposition when unfavorable interactions between the components bring the system into the thermodynamically unstable region of the phase diagram [39]. However, spinodal decomposition is a spontaneous process and phase separation will start instantly when entering the unstable region (solvent quench) [40], while crystallization requires the formation of nuclei [38]. BHJ samples are often subjected to annealing, which alters the film morphology.

If structure formation is dominated by crystallization and the system can be described by an eutectic phase diagram, calorimetry is a suitable method to map the phase boundaries. Müller et al. constructed phase diagrams from differential scanning calorimetry (DSC) thermograms for the binary poly(3-hexylthiophene-2,5-diyl) (P3HT)/phenyl-C_61_-butyric acid methyl ester (PC_61_BM) system. They found that the best solar cell performance is obtained using the blend composition of the hyper-eutectic point, PC_61_BM being the excess compound [41].

The construction of phase diagrams from DSC and the identification of eutectic points and amorphous regions has been also successfully applied to ternary organic photovoltaic (OPV) blend systems, as it delivers a rather good indication if a second component hampers the formation of crystalline domains. Crystalline domains have been identified as essential for charge transport. Works on the ternary blend of poly[2,1,3-benzothiadiazole-4,7-diyl[4,4-bis(2-ethylhexyl)-4H-cyclopenta[2,1-b:3,4-b’]dithiophene-2,6-diyl]] (PCPDTBT), P3HT and PC_61_BM was introduced by Li and co-workers [42]. They found that even addition of small amounts of the amorphous polymer (PCPDTBT) leads to a decrease of the ternary blend crystallinity, and that the corresponding devices show declined performance.

In this paper, we base nanostructure formation on spinodal decomposition as described by the theory of Flory and Huggins [43]. Molecular weight and interaction between the different components greatly determine the phase structure (miscibility, segregative phase separation and assertive phase separation, e.g. complex coacervation) [44,45]. For polymer electrolytes, it has been shown both theoretically and experimentally that control over the phase behavior can be gained via ionic interactions. The incorporation of charged substituents into polymer chains is greatly enhancing polymer miscibility. Dissociated counterions increase the entropy of mixing and circumvent phase separation [46,47]. The sensitizer used in our studies is a cationic cyanine dye. Cyanine dyes have been used as OPV materials for a number of interesting properties [48–50]. Cyanine dyes can here be a valuable model system as miscibility can be tuned via exchange of the counterion.

Exploiting this effect, we compare the photovoltaic performance of two ternary systems where we add a cyanine dye with different counterions. While there is a minor influence of the counterion on the optoelectronic properties of the dye [50], when mixed with other components, huge differences in morphology are observed. The underlying binary blend system is the polymer PBDTTT-C (poly[(4,8-bis-(2-ethylhexyloxy)-benzo(1,2-b:4,5-b′) dithiophene)-2,6-diyl-alt-(4-(2-ethylhexanoyl)-thieno[3,4-b]thiophene-)-2–6-diyl)]) blended with PC_70_BM [51]. The third component is the cyanine dye (2-[2-[2-chloro-3-[2-(1-ethyl-1,3-dihydro-3,3-dimethyl-2H-indol-2-ylidene)ethylidene]-1-cyclohexen-1-yl]ethenyl]-1-ethyl-3,3-dimethyl-3H-indolium with the counterions (OC-6–11-Δ)-tris[3,4,5,6-tetrachloro-1,2-benzenediolato(2-)-κO1, κO2]phosphate(1-) (Cy7T) or hexafluorophosphate (Cy7P). Dyes with the two counterions have been reported to strongly segregate from PC_61_BM (Cy7P) or be compatible with PC_61_BM (Cy7T) [52]. The absorption regions of the three materials complement each other and can be easily identified via UV-Vis spectroscopy and external quantum efficiency (EQE) measurements. Highest occupied molecular orbital (HOMO) and lowest unoccupied molecular orbital (LUMO) levels of the materials are distinct and make only certain electron- and energy transfer processes likely. Cy7 can be selectively removed from the thin films, which allows a rough reconstruction of the phase morphology with atomic force microscopy (AFM) and UV-Vis spectroscopy. Combining this information gives us valuable insight into the interplay between phase behavior and solar cell performance.

## 2. Experimental section

### 2.1. Device fabrication

The photovoltaic performance of the different ternary blend systems was investigated in an inverted architecture of ITO/TiO_2_/active layer/MoO_3_/Ag, where ITO stands for indium tin oxide. PBDTTT-C was purchased from Solarmer Materials Inc., and PC_70_BM (99%) was purchased from Solenne BV. Cy7T and CyP were synthesized in our laboratory as described elsewhere [53]. Titanium isopropoxide (99.999%), 1,2-dichlorobenzene (DCB) and 1,8-diiodooctane (DIO) were purchased from Sigma-Aldrich, and MoO_3_ (99.9995%) from Alfa Aesar. All chemicals were used as received.

Indium tin oxide-coated glass substrates (140 nm, resistivity 20 Ω/sq) were sequentially cleaned in acetone, ethanol, detergent and de-ionized water. Cleaned ITO substrates were used to deposit TiO_2_ films to form an electron transporting layer via a sol-gel process, using titanium isopropoxide as precursor [15,54]. Spin coated TiO_2_ films were heated to reach 460 °C in 3h in air, held for 2h, and then cooled down to room temperature. The thickness of the film was 35 nm. TiO_2_ coated substrates were heated inside a glovebox for 10 min at 140 °C before spin coating the different active layers. PC_70_BM and PBDTTT-C were dissolved in DCB, and the weight ratio of to PC_70_BM to PBDTTT-C was kept constant at 1.5: 1.0 (PC_70_BM: PBDTTT-C = 15 mg: 10 mg in 1 ml of DCB). 3% volume ratio of DIO was then added to the mixture and stirred before use [15,55]. The binary blends were doped with Cy7P or Cy7T as specified. Abbreviations for the blends (Cy7P01, Cy7P03, Cy7P05, Cy7T01, Cy7T03, Cy7T05) are defined in Table 1. Cy7 refers to both Cy7P and Cy7T. The binary and ternary blends were spin coated with a spin speed of 900 rpm for 60 s in a nitrogen filled glove box. Devices with different amounts of Cy7 showed consistent thicknesses of 95 ± 5 nm.

**Table 1.** Summary of solar cell parameters of the ternary blend PC_70_BM: Cy7: PBDTTT-C with different contents of Cy7T and Cy7P and short names for the blends. The irradiance was 91 mW cm^−2^. J_sc_ values are extracted from the J-V curves as well as from integrating the EQE (in brackets) .

Amount [wt%]	V_oc_ [V]	J_sc_ [mAcm^−2^]	FF [%]	PCE [%]
PC_70_BM: PBDTTT-C				
1.5: 0.0: 1.0	0.7	12 (11.9)	65	5.2 ± 0.6
PC_70_BM: Cy7T: PBDTTT-C				
1.5: 0.1: 1.0 (Cy7T01)	0.68	13 (12.0)	57	5.1 ± 0.2
1.5: 0.3: 1.0 (Cy7T03)	0.68	12 (11.3)	52	5.0 ± 0.2
1.5: 0.5: 1.0 (Cy7T05)	0.67	10 (9.0)	49	3.9 ± 0.2
PC_70_BM: Cy7P: PBDTTT-C				
1.5: 0.1: 1.0 (Cy7P01)	0.58	9.2 (8.2)	49	2.4 ± 0.3
1.5: 0.3: 1.0 (Cy7P03)	0.50	6.8 (5.0)	45	1.4 ± 0.2
1.5: 0.5: 1.0 (Cy7P05)	0.39	3.6 (2.9)	36	0.6 ± 0.1

Finally, a 20 nm MoO_3_ hole conducting layer and 80 nm thick silver electrodes were deposited by thermal evaporation at a pressure < 5 × 10^−6^ mbar. Silver was evaporated through a shadow mask to deﬁne eight solar cells on each substrate with active areas of 3.1 and 7.1 mm^2^.

### 2.2. Characterization

A calibrated xenon lamp based solar simulator (Spectra-Nova) with a simulated AM1.5G solar radiation of 91 mWcm^−2^, equipped with a Keithley 2400 source/measure unit was used to measure the current density – voltage (J-V) characteristics of the solar cells. The light intensity was calibrated with a standard silicon solar cell. The devices were sealed in a vacuum-tight box with current feedthroughs and an optical window that reflected 9% of the calibrated light intensity [15]. The external quantum efficiency (EQE) was measured using a quartz tungsten halogen lamp operating at 100 W in combination with a 150 mm focal length Czerny-Turner monochromator (SpeQuest). The monochromatic light intensity was calibrated using a silicon detector. The morphology of the organic layers was scanned with an AFM (NanoSurf Mobile S in tapping mode). Films thicknesses were determined with a profilometer (Ambios XP1). Conventional absorption spectra of the different organic films were measured on a Varian Cary 50 UV-vis spectrophotometer. NanoIR composition maps were collected with a nanoIR2-FS setup from Anasys Instruments (Santa Barbara, CA) equipped with a quantum cascade laser. I-V curves were fitted using a routine described by us earlier [56].

### 2.3. Simulations

Modeling routines were written in the Python programming language. The code used to plot Figure 6(d,e) is available from the Supporting Information. The plots were generated using the python-ternary library [57]. First, a ternary phase diagram with a composition point grid of arbitrary density is defined. Taking into account the estimated size of the components and their interaction parameters, the free energy at every point is calculated following the Flory-Huggins model [58]. The spinodal regions were found by comparing the energy of a point with the average energy of all pairs of opposite direct neighbors [59]. If the energy of one of those pairs is lower than the one of the point of interest, the total energy of the system can be lowered by a spontaneous separation into the neighboring compositions and therefore, the point is marked as unstable. The impact of the dissociated counterions is modeled by adding an entropy term to the Flory–Huggins model as suggested by Hellebust et al. [60].

**Figure 1.**
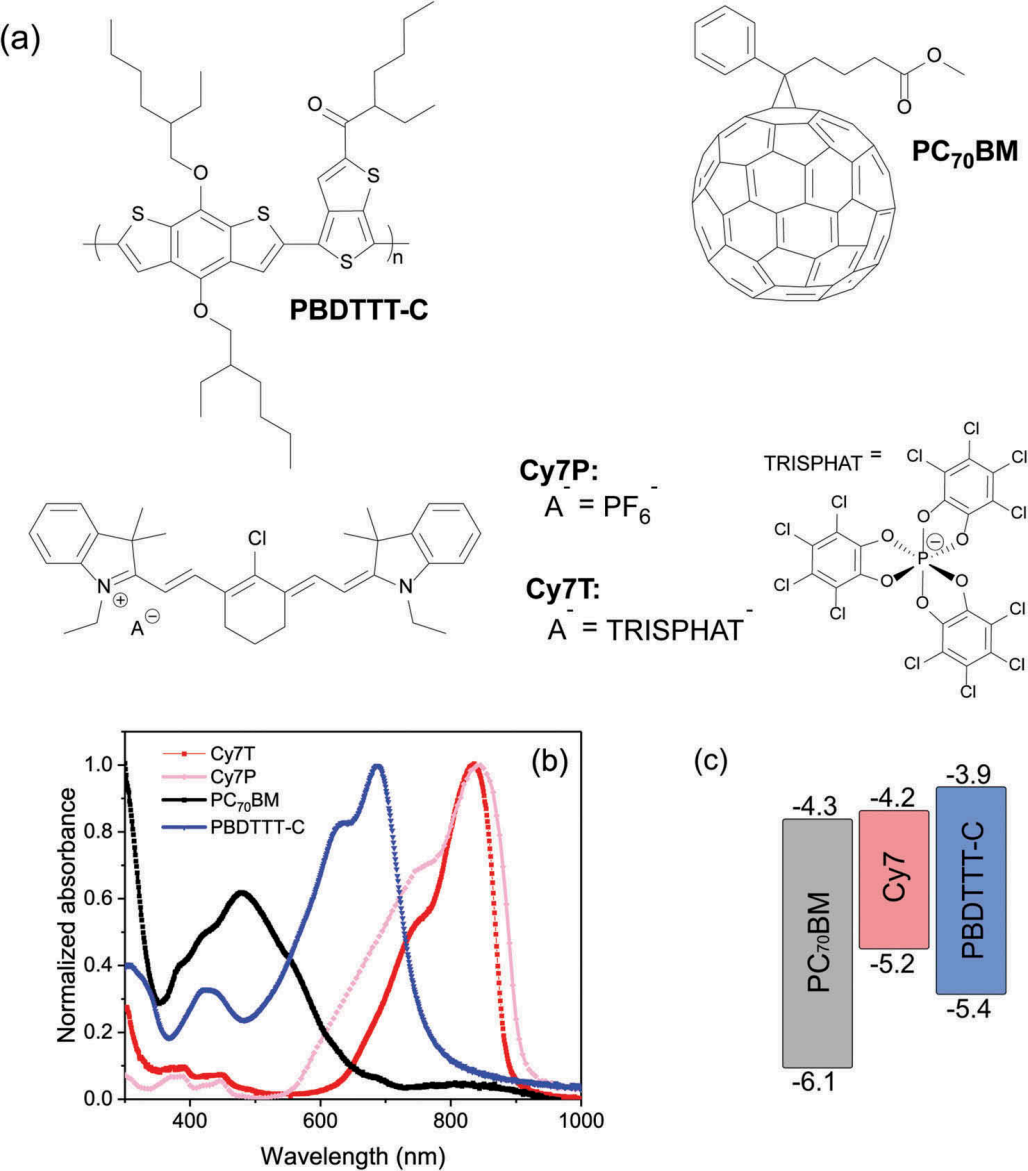
(a) Chemical structures of PBDTTT-C, PC_70_BM and the dye Cy7 with counterions hexafluorophosphate (Cy7P) and TRISPHAT (Cy7T). (b) Normalized absorbance spectra (films on glass) of pristine PBDTTT-C, Cy7T, Cy7P and PC_70_BM. (c) Energy band diagram of PC_70_BM, Cy7 and PBDTTT-C.

**Figure 2.**
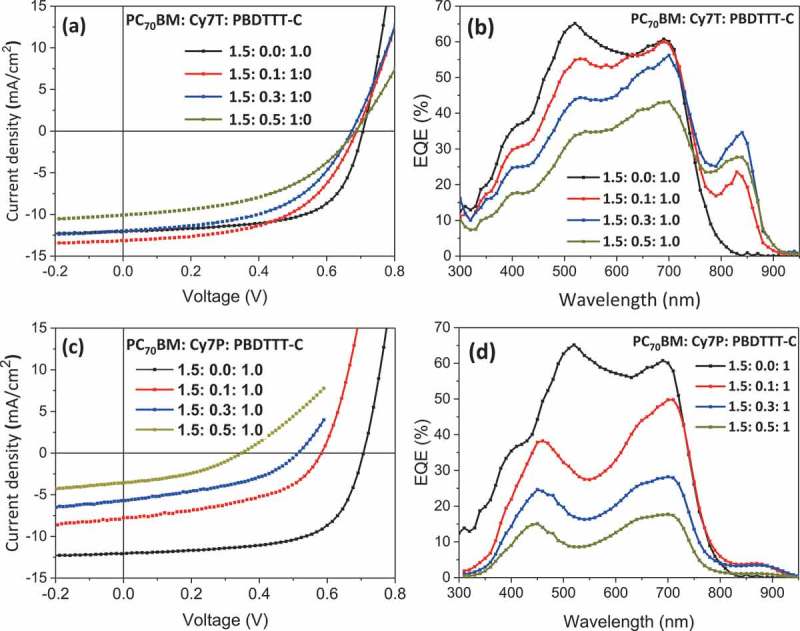
(a), (c) J-V characteristics and (b), (d) EQE spectra of devices with the active layer PC_70_BM: Cy7: PBDTTT-C with different weight fractions of Cy7T and Cy7P under simulated AM 1.5 illumination at 91 mWcm^−2.^

**Figure 3.**
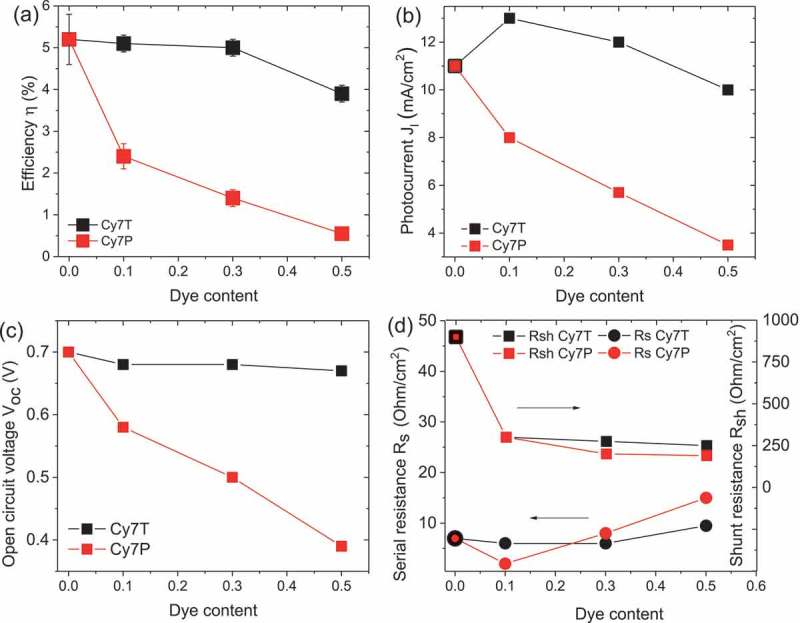
Solar cell parameters extracted from the J-V curves. (a) Efficiency η. (b) Photocurrent J_l_. (c) Open circuit voltage V_oc_. (d) Shunt R_sh_ and serial resistance R_s._

**Figure 4.**
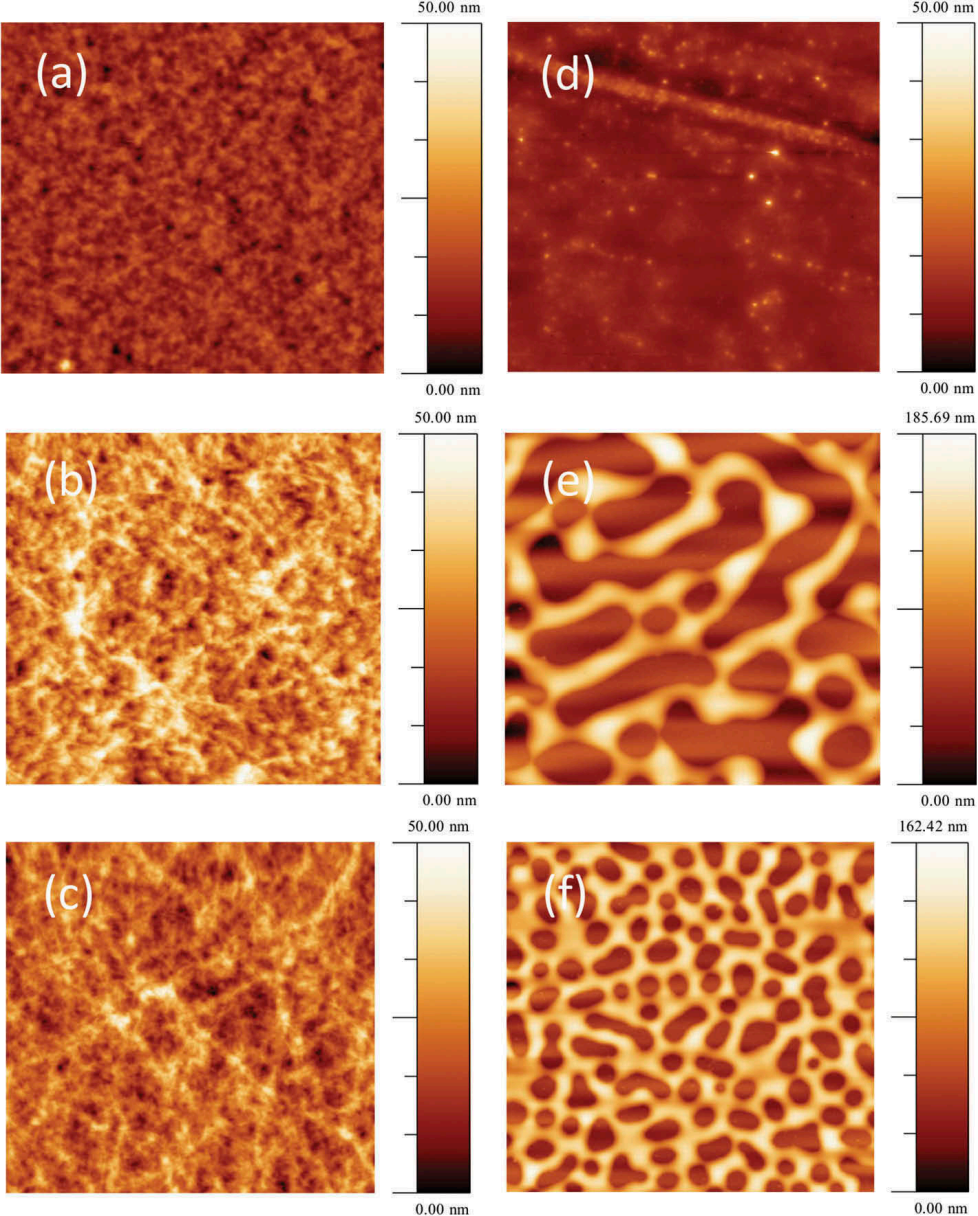
Morphology of active layer films spin coated onto glass: (a) PC_70_BM: PBDTTT-C 1.5: 1.0, Image size: 10 μm × 10 μm. (b) Cy7T03 (c) Cy7T05. Image size: 5 μm × 5 μm. (d) Cy7P01. (e) Cy7P03. (f) Cy7P05. Image size: 10 μm × 10 μm.

**Figure 5.**
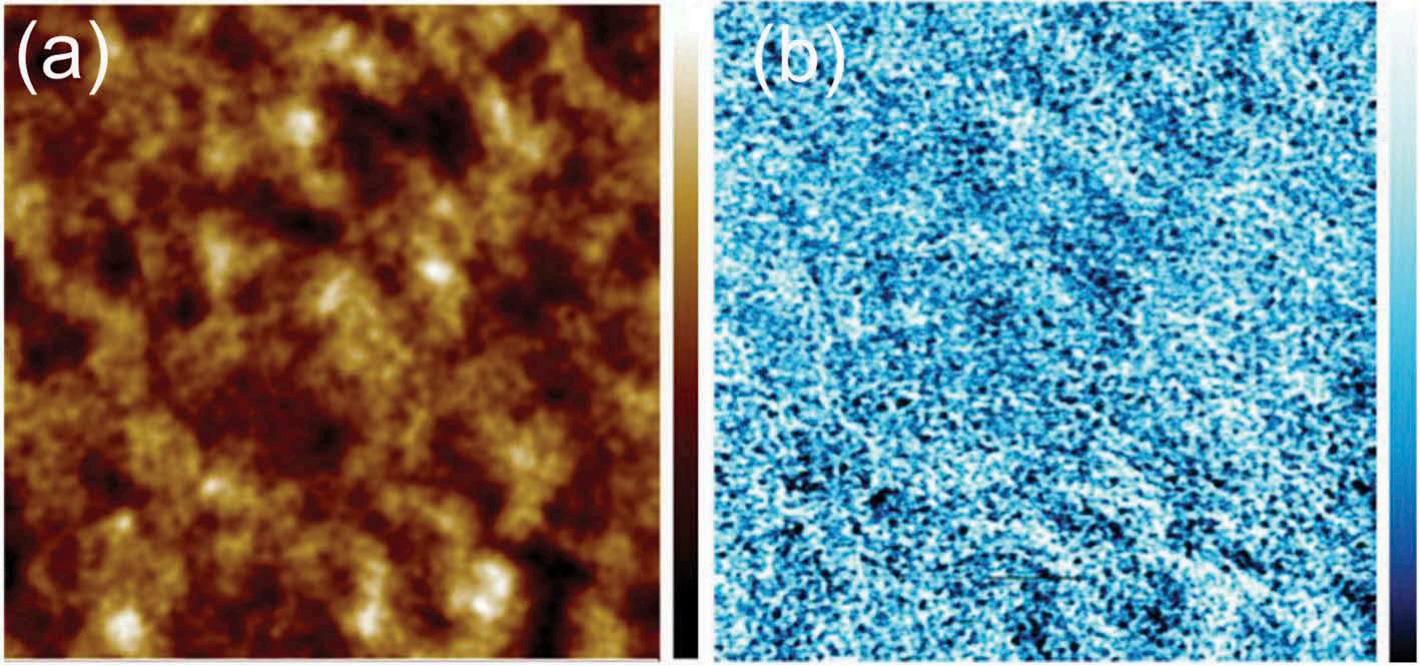
Tapping AFM-IR image of a ternary blend of PC_70_BM: Cy7T: PBDTTT-C (Cy7T05). (a) Topography, (b) IR image collected at 1736 cm^−1^ (PC_70_BM). Image size: 5 μm × 5 μm. The data scale of the height is 50 nm, the data scale of the IR signal is 0.5 mV.

**Figure 6.**
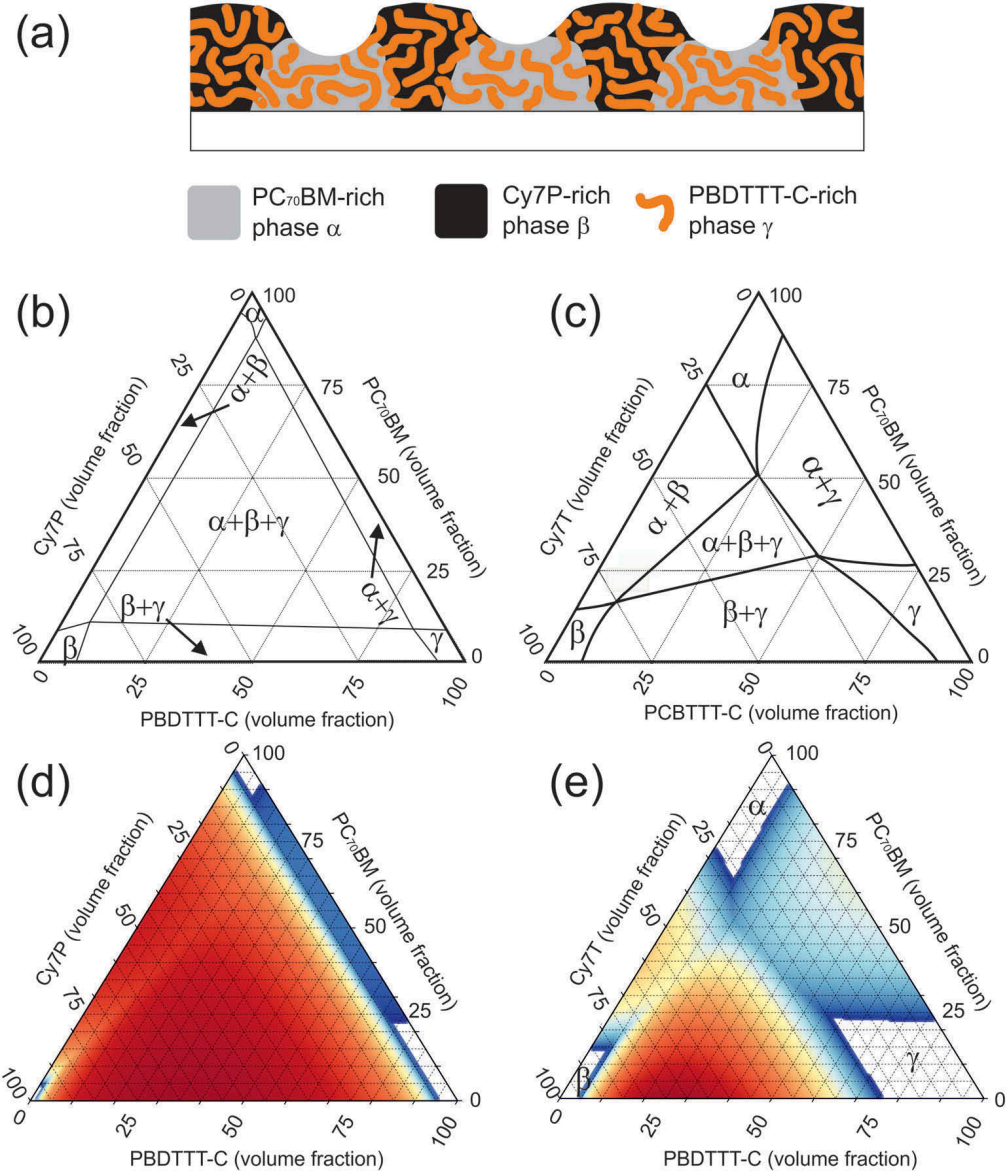
(a) Sketch of the ternary blend morphology of PC_70_BM: Cy7P: PBDTTT-C. (b) and (c) Sketch of the ternary phase diagram of PC_70_BM: Cy7P: PBDTTT-C and PC_70_BM: Cy7T: PBDTTT-C, respectively. (d) and (e) Simulations of the ternary phase diagrams. For PC_70_BM: Cy7P: PBDTTT-C a fully associated counterion was assumed (d). For PC_70_BM: Cy7T: PBDTTT-C 60% of the ions were assumed to be dissociated (e). All other parameters were kept the same for both systems: N_1_ = 15, N_2_ = 15, N_3_ = 50, N_4_ = 5, χ_12_ = 0.8, χ_23_ = 0.8, χ_13_ = 0.16.

## 3. Results

### 3.1. Photovoltaic properties

Chemical structures of PBDTTT-C, Cy7T, Cy7P and PC_70_BM are shown in Figure 1(a), and their thin film UV-Vis absorption spectra are presented in Figure 1(b). The maximum absorption wavelengths of PBDTTT-C, Cy7T and Cy7P are at 688, 837 and 844 nm, respectively. In this respect, Cy7T or Cy7P dyes expand the film absorption into the NIR. The absorbance spectrum of Cy7P is broader than the spectrum of Cy7T. Broadening is frequently caused by intermolecular interactions of molecules that show random separation and orientation toward each other [61]. Drastic changes are attributed to dye aggregation, as visible in the blue-shifted shoulder [62]. We have shown before that the counterion has a strong effect on the packing of the molecules [50]. UV-absorption spectra of the blend films are shown in Figure S1.

The HOMO/LUMO levels of PBDTTT-C, Cy7 and PC_70_BM are 5.4/3.9 eV [63], 5.2/4.2 eV [53] and 6.1/4.3 eV [64], respectively, summarized in Figure 1(c). The energy levels allow speculations at which interfaces exciton dissociation can take place and through which phases charge can be transported to the electrode. Taking the pure single components, the system does not show a cascade energy level alignment that would make charge transfer throughout the ternary system a possible mechanism. Rather a parallel-linkage model applies, where PC_70_BM acts as acceptor for either PBDTTT-C or Cy7T. When Cy7T is in contact with PBDTTT-C, energy transfer from the polymer to the dye may occur.

As reference device a binary blend of PC_70_BM: PBDTTT-C was optimized with respect to thickness and composition. The control device showed a short circuit current density (J_sc_) of 12 mA cm^−2^, an open circuit voltage (V_oc_) of 0.7 V, a fill factor (FF) of 65% and a power conversion efficiency (PCE) of 5.2% at 91 mW cm^−2^. Extrapolated to full sun, this corresponds to a PCE of 6%, which is comparable to the literature values [51]. The EQE spectrum of the reference device showed a combined photoresponse of both the donor PBDTTT-C and the acceptor PC_70_BM, in accordance with the energy levels in Figure 1(c) and consistent with a BHJ morphology, enabling oxidative and reductive photocurrent generation (Figure 2(b)). The EQE maximum at 528 nm is actually red-shifted by 50 nm from the absorption maximum of PC_70_BM. This is due to a thickness related filter effect, which is discussed in the Supporting Information (Figure S2). Depending on the counterion, very different responses were visible in the EQE and the J-V curves (Figure 2). The weight ratio of PC_70_BM to PBDTTT-C was kept constant at 1.5: 1.0 [15]. Nomenclature and exact sample composition are summarized in Table 1 and Table S1.

The addition of a dye contributed to photocurrent only in the Cy7T blends. The EQE in the fullerene: polymer absorption region decreased with increasing dye content for both dyes. In the Cy7T blends, small dye additions only decreased the EQE around the peak at 528 nm assigned to PC_70_BM. In the Cy7P blends, a drastic decrease was observed even for small dye addition, and the EQE peaks shifted to the positions observed for thinner films.

Basic photovoltaic performance parameters are summarized in Table 1 and Figure 3. PCE, J_sc_, V_oc_ and fill factor decreased slightly for Cy7T, while they dropped dramatically with dye content for the Cy7P blend (Figure 3). For Cy7T01 the additional absorption in Cy7T also led to an increase in J_sc_ (Table 1). The J-V curves of the samples under illumination were fitted with the formula for an equivalent circuit diagram of a photodiode (extended Shockley equation) [65],

(1) $$J=-J_{l}+J_{0}\left\{\exp\left[\frac{e}{nkT}\left(V-R_{S}J\right)\right]-1\right\}+\left(\frac{V-R_{S}J}{R_{Sh}}\right)$$

with light generation current J_l_, series resistance R_s_, shunt resistance R_sh_, reverse saturation current J_0_ and diode ideality factor n as fitting parameters. We report fitting parameters of the J-V curves recorded under illumination as only these values reflect all generation and recombination channels. These values are plotted in Figure 3 and S3.

While almost all characteristic values differed from system to system, shunt and series resistance R_sh_ and R_s_ behaved the same for both counterions: the smallest addition of dye led to a drop of the shunt resistance R_sh_ to about one quarter of the initial value. The series resistance R_s_ did remain almost constant, showing that addition of dye does not affect the internal serial resistance of the active layer. Less clear were the trends in reverse saturation current and ideality factor (Supporting Information, Figure S3).

### 3.2. Film morphologies

The binary mixture PC_70_BM: PBDTTT-C underlying the ternary blends is described in previous work [15,51]. The critical blend phase separates into an interconnected network of PC_70_BM and PBDTTT-C rich phases. It is generally believed that this two-phase morphology represents a near ideal phase structure with a sufficiently large interface area for exciton dissociation and interconnected charge transport channels for electrons as well as for holes. The characteristic length-scale of the domains resulting from this phase separation process is in the range of 10 nm. An AFM image of such a film did not show a large topography contrast (Figure 4(a)) [15]. Adding Cy7T to the binary blend did not alter the surface topography drastically, for the Cy7T05 blend the films showed fibrous features (Figure 4(a-c)). Differently, the PC_70_BM: Cy7P: PBDTTT-C system showed large topography contrasts in the AFM, indicative for liquid-liquid dewetting (Figure 4(d-f)).

We have reported earlier that Cy7T shows miscibility with PC_61_BM [52]. It is likely that also in the ternary blend the dye does not form a separate dye-rich phase but blends into the fullerene and polymer. To substantiate that claim, the blend film Cy7T05 was probed with the element-sensitive scanning probe technique nanoIR^TM^ (Figure 5). A PC_70_BM map was obtained by measuring IR absorption at 1736 cm^−1^, characteristic for the carbonyl absorption (C = O) of PC_70_BM (Figure 5(b)) [66]. NanoIR spectra at different locations of the sample are shown in Figure S5. The image unambiguously proved that a nanoscale morphology is dominating, even though a significant amount of dye had been added to the sample. To remove any doubt that the contrast in IR signal does not have its origin in thickness variations, the image was also scanned at a wavelength off any absorption band (Figure S5).

Differently, Cy7P is highly incompatible with PC_61_BM, and formed large liquid-liquid dewetting morphologies [67]. The features are self-similar to the structures we were reporting for the ternary PC_70_BM: Cy7P: PBDTTT-C blend. We can thus assume that the immiscibility of dye and fullerene dominate the ternary blend behavior and follow a similar dewetting process as reported before [67]. During film formation, PC_70_BM and Cy7P segregated to substrate and film interface, respectively, while PBDTTT-C remained more or less evenly distributed throughout the film. Within this layered structure, PBDTTT-C phase separated from both, Cy7P and PC_70_BM, but on a much smaller length scale due to the lower diffusivity of the high molecular weight polymer. Afterwards, the two layers phase separated by liquid-liquid dewetting, leading to the large scale laterally separated structures shown in Figure 4(e) and 4(f). The elevated regions correspond to Cy7P: PBDTTT-C blends, while the recessed regions to the PC_70_BM: PBDTTT-C blends (Figure 6(a)) [67]. This assumption is supported by the observation that with increasing dye content the fraction of elevated areas increases. For low dye content, the laterally segregated regions did not result in a topographical contrast (Figure 4(d)). The exact phase morphology of the sub-phases was difficult to assess, but we may assume that in the final morphology two complementary BHJs coexist (Figure 6(a)).

### 3.3. Ternary phase diagrams

Phase diagrams are a powerful tool to understand film morphologies. In the Cy7P systems, all three components were highly incompatible and a diagram could look like the diagram depicted in Figure 6(b). The composition space is dominated by a three phase region where an α¸ β and γ phase coexist. The α¸ β and γ phase were defined as PC_70_BM, dye and PBDTTT-C-rich phases, respectively. In the Cy7P system, we assumed that the phases are almost pure components. Nonetheless, the equilibrium phase diagrams neither revealed information how these phase domains are distributed within the sample, nor which length scales are involved. It should be noted that the observed phase morphology is not necessarily an equilibrium morphology, while the phase diagram itself represents thermodynamic equilibrium. Given that large scale structures are observed in the Cy7P system, we can assume that the phase composition is in equilibrium.

For the Cy7T system, additional insights up to the construction of a full ternary phase diagram were achieved with UV-vis spectroscopy. Here the selective solubility of the dye in acetonitrile was exploited. The films were immersed in acetonitrile and AFM images and UV-vis spectra were compared for untreated and treated films. This way the β phase (dye rich phase) was removed from the film. Remaining dye must be dissolved in the α and γ phase which is not soluble in acetonitrile. We assumed that no small dye domains were encapsulated by a different phase. In very thin films this is a reasonable assumption. The procedure is described in detail in the Supporting Information (Figure S4). With the obtained data, a rough course of the ternary phase diagram was drawn (Figure 6(c)). A compatibilizing third component (dye) expanded the existing single- and two-phase regions at cost of the three-phase region. We proposed compatibilization by an increased mixing entropy via the introduction of a counterion that dissociates easily from the chromophore. The radius of the TRISPHAT counterion is much larger than the radius of PF_6_^−^. It has been shown theoretically and experimentally that the dissociation constant increases with radius [68]. To give evidence for such an operation principle, we calculated the phase diagram based on a free energy model following the theory of Flory and Huggins [58].

### 3.4. Calculated phase diagrams

Various approaches extended the Flory–Huggins theory to ionic compounds. Khokhlov and Nyrkova dealt with ions by introducing an effective molecular weight [69], we here followed a description of Hellebust et al. where the ions are treated as a fourth component [60]. In this model the free energy of mixing per lattice site is given by:

(2) $$\Delta G_{mix}\;=\;kT[(\phi_{1}/N_{1})ln\phi_{1}\;+\;(\phi_{2}/N_{2})ln\phi_{2}\;+\;(\phi_{3}/N_{3})ln\phi_{3}\;+\;\phi_{4}\ln N_{4}+\;\chi_{12}\phi_{1}\phi_{2}+\;\chi_{13}\phi_{1}\phi_{3}+\;\chi_{23}\phi_{2}\phi_{3}]$$

where ϕ_i_ are the volume fractions of each component, N_i_ are the number of lattice sites each component occupies and χ_ij_ are the Flory-Huggins interaction parameters. In our study, the compounds were indexed as follows: index 1 = PC_70_BM, 2 = Cy7, 3 = PBDTTT-C and 4 denoted the counterion. In Hellebust et al.’s model, the interaction between the counterion and the other components are neglected. This can be justified for two reasons: the entropic part plays a more important role and at high ionic content electrostatic screening is at play.

A computational method to generate such a diagram is also described in appendix A of ref [60]. We followed a computational less challenging approach described by Horst [59]. The impact of the dissociated counterions was modeled by adding an entropy term to the Flory-Huggins model as suggested by Hellebust et al. The Flory-Huggins parameters were taken as fitting parameters, with the intention to reproduce the phase diagrams shown in Figure 6(b) and 6(c) by varying only the degree of ion dissociation. The results are shown in Figure 6(d) and 6(e) for the counterion associated to the dye and the case where 60% of the counterions are dissociated, respectively, while all other variables were kept constant. Colors represent unstable points; the color code indicates the spinodal driving force, defined by the free energy difference between a point and the lowest average of its neighboring pairs. Red represents a strong spinodal driving force, while blue stands for a weak free energy difference. The simulations reproduced the diagrams constructed from our experimental data reasonably well, especially the expansion of all single- and two-phase regions was obvious.

## 4. Discussion

### 4.1. PC_70_BM: Cy7p: PBDTTT-C system

**Figure 7.**
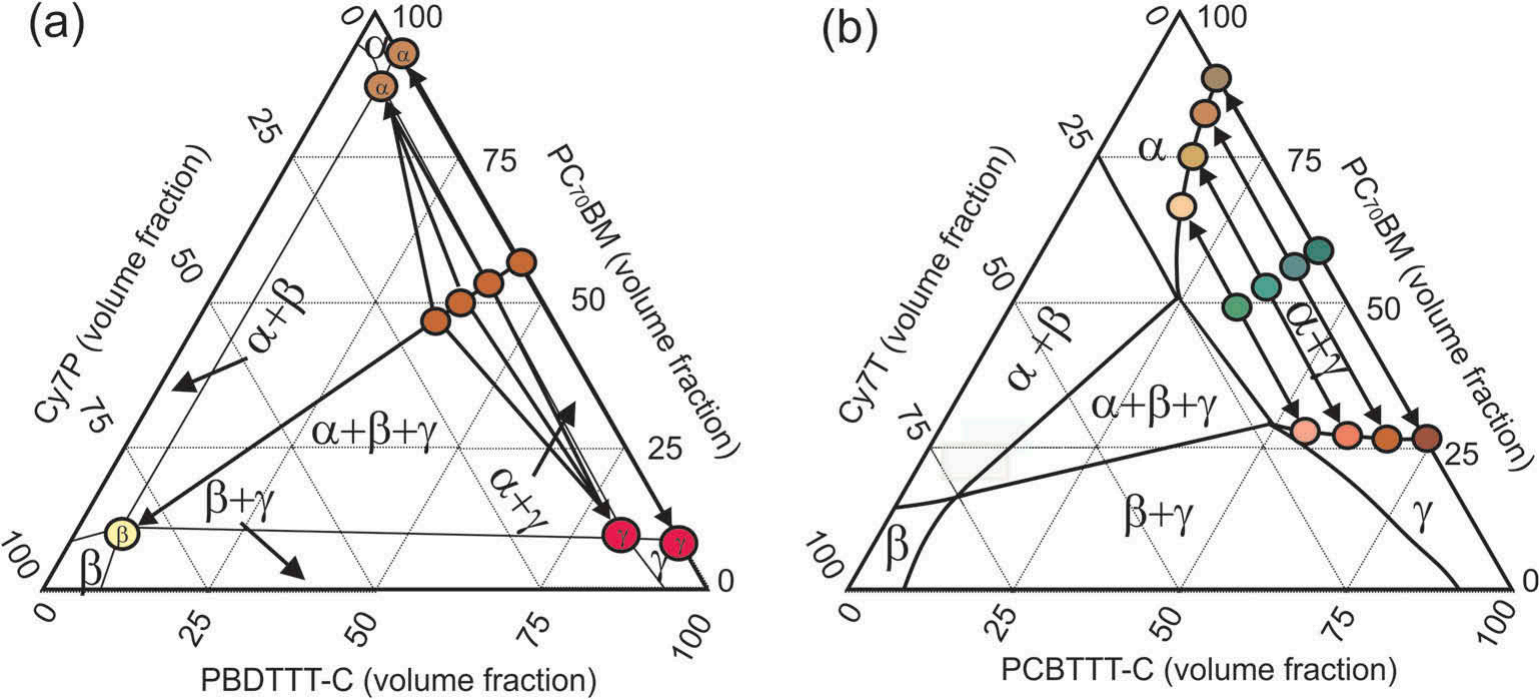
Schematic of a ternary phase diagram of PC_70_BM: Cy7: PBDTTT-C with (a) Cy7P and (b) Cy7T. The starting compositions of the blends with increasing Cy7 contents 0.1, 0.3 and 0.5 are represented in the diagrams as well as the compositions of the final phases. The ternary blend with Cy7P blend separates into three phases α¸ β and γ. The ternary Cy7T blends separate into two phases α and γ whereby the compositions are determined by the binodal.

We here correlate solar cell data presented in the first section to the phase morphology. For the blends doped with Cy7P, all investigated ternary blend compositions phase separated into α¸ β and γ phases. This is visualized in Figure 7(a). Even the smallest addition of dye brought the blend from a two-phase into a three-phase system.

These three phases are distributed such that two laterally separated and different BHJs formed. An interpenetrated network of β/γ-phases dewetted from the α/γ-phase network, building the large scale framework for a hierarchical phase structure (Figure 6(a)). With this model we can explain all characteristics of the photovoltaic behavior: (1) the dye did not contribute to the EQE (Figure 2(d)) because the sub-part of the film forming the Cy7P: PBDTTT-C BHJ cannot contribute to photocurrent. Excitons generated in the dye phase cannot dissociate into free charge carriers at the polymer interface. Excitons generated in the PBDTTT-C can at best transfer to the dye by energy transfer (Figure 1(c)). (2) The EQE changed shape and resembled the EQE of the thinner film EQE of the binary PC_70_BM: PBDTTT-C blend (Figure S2). This is consistent with the claim that the PC_70_BM: PBDTTT-C BHJ formed the thinner film sections (Figure 6(a)). (3) That also explains that a film with micron sized lateral and topographical features still delivered efficiencies of 1.4%. (4) With increasing dye content the photocurrent was decreasing further because dye is binding parts of PBDTTT-C, with increasing dye content the fraction of polymer in the PC_70_BM: PBDTTT-C sub-BHJ was decreasing. For that reason the EQE also decreased more strongly in the polymer part of the spectrum. (5) Decrease of V_oc_: with the composition change of the sub-cell, the BHJ morphology transformed from an interconnected network into a PC_70_BM matrix with isolated PBDTTT-C domains. In such a morphology, photogenerated charges are trapped inside the domains, favoring recombination. Consequentially V_oc_ was lowered and the reverse saturation current increased (Figure 3(c) and S3(a)). Morphology and solar cell parameters both suggested that two factors degrade solar cell performance upon adding C7P: diode currents increased and the shunt resistance dropped because of thinning of sections of the film.

### 4.2. PC_70_BM: Cy7t: PBDTTT-C system

The situation is quite different in the PC_70_BM: Cy7T: PBDTTT-C system, visualized in Figure 7(b). All samples Cy7T01, Cy7T03 and Cy7T05 fell into the two-phase region defined by the phase separation of PC_70_BM and PBDTTT-C (α + γ phase). Here, the dye did not form its own phase, but was intermixed (blended) into other phases. The importance of intermixed phases for photovoltaics has been discussed before for PC_61_BM, which showed considerable miscibility with donor polymers [70]. In BHJ solar cells charges are generated, but also recombine via charge transfer states. In an intermixed phase charge generation is facilitated, while charge extraction favors pure phases. Thus, in a binary blend optimal phase compositions exist, which compromise between charge generation and charge extraction [71]. Following the binodal in the ternary phase diagram in Figure 7(b), it can be generalized that the addition of a third component is a powerful mean to direct the degree of intermixing (e.g. solubility of PCBM within the polymer). This strategy had been successfully applied to photochemically inactive materials [72]. For the Cy7T system that meant: the more dye was added, the higher the amount of minority components (PC_70_BM & Cy7T in PBDTTT-C and Cy7T & PBDTTT-C in PC_70_BM). To understand how that blending behavior is influencing electrical properties, we measured charge generation and extraction efficiency and hole mobility and electron mobility (Supporting Information, Figures S7 and S8, Table 2) [73,74]. The product of charge generation and extraction efficiency (Figure S7) showed for all ternary blends (Cy7T01, Cy7T03, Cy7T05) a similar behavior that differed from the binary blend. While the binary blend reached about 80% of the saturation current at V_eff_ = 0.1 V and showed a sharp transition toward saturation, in the ternary blends the current increase was much slower and the transition toward saturation less distinct. Interestingly, also for the shunt resistance the behavior differed from binary to ternary blends, but was almost identical for all doped samples.

**Table 2.** Summary of hole and electron mobilities in ternary blends PC_70_BM: Cy7: PBDTTT-C.

	Hole mobility [cm^2^V^−1^ s^−1^]	Electron mobility [cm^2^V^−1^ s^−1^]
PC_70_BM: PBDTTT-C		
1.5: 0.0: 1.0	1.04 × 10^−3^	9.5 × 10^−4^
PC_70_BM: Cy7T: PBDTTT-C		
1.5: 0.1: 1.0 (Cy7T01)	1.02 × 10^−3^	9.6 × 10^−4^
1.5: 0.3: 1.0 (Cy7T03)	6.1 × 10^−4^	6.0 × 10^−4^
1.5: 0.5: 1.0 (Cy7T05)	5.9 × 10^−4^	1.2 × 10^−4^

The hole and electron mobilities of the binary blend and Cy7T01 ternary blend were quite similar; lower, but also similar were hole mobilities of Cy7T03 and Cy7T05 ternary blends and the hole mobility in a Cy7T03 film. The electron mobility in the Cy7T05 blend dropped by almost one order of magnitude (Table 2).

The fact that mobilities did not change over orders of magnitude gave us confidence that the parameters extracted from J-V curve fitting can be compared to each other [75]. We anyway do not claim to infer correct information about the recombination process, but reveal a coarse tendency of physical parameters upon dye addition to correlate them to changes in morphology.

The trend in solar cell parameters is now evident from the phase diagrams: in the minority component enriched phases, charge generation was increasing, but their role as exclusive electron- or hole-transport layers suffered. The role of shunt currents over diode currents increased as also seen in the J-V curve fits. It is also apparent from this picture that the more negative the bias, the more charges can be extracted.

The rather moderate decrease in mobilities also indicated the preservation of the binary morphology.

To fully understand the EQE behavior, the photovoltaic response was investigated in the remaining two-phase regions (α + β and β + γ) as well as in the three-phase region (α + β + γ) and the α–phase (Supporting Information, Figure S9). From the data we can understand why at low dye doping (Cy7T01) the contribution of PBDTTT-C was not affected, while photogeneration from PC_70_BM was lowered instantly (Figure 2(b)). Dye is blended into both phases, given the very similar HOMO and LUMO levels of Cy7T and PBDTTT-C, dye and polymer complement each other in one phase. On the other side, dye blended into the fullerene acts as an exciton trap, lowering the PC_70_BM contribution to the EQE instantly.

## 5. Conclusions

We showed that ternary phase diagrams are a powerful tool for understanding the photovoltaic behavior in ternary blend systems. In a typical ternary system for photovoltaic applications, a third sensitizing component is added to an underlying well performing binary system. It is generally assumed that a third phase forms nanometer scale domains that are distributed within the binary blend system. With our two model systems we demonstrated that the description of ternary systems can be much more complicated. Next to the composition space where the system separates into three phases, a ternary diagram is characterized by six more composition regions: the stable α¸ β and γ regimes plus three regions where the system separates into two phases. Especially, a phase in a ternary system is composed of the majority component plus two minority components, an important, often overlooked aspect when discussing phases in phase separating systems.

Last but not least, we introduced a very efficient method to control phase compatibility via ionic interactions. In current work, we are searching for a counterion which shows intermediate mixing behavior.
