# Supplementary material for: Insights into photovoltaic properties of ternary organic solar cells from phase diagrams
Source: Sci Technol Adv Mater. 2018 Sep 25;19(1):669–82. doi: 10.1080/14686996.2018.1509275 (PMC6161617; doi:10.1080/14686996.2018.1509275)
Supplement: Supplemental Material [file TSTA_A_1509275_SM2618.docx]

Supporting Information

Insights into photovoltaic properties of ternary organic solar cells from phase diagrams

Mohammed Makha*, Philippe Schwaller, Karen Strassel, Surendra B. Anantharaman, Frank Nüesch, Roland Hany, Jakob Heier*

**SAMPLE LABELS, WEIGHT AND VOLUME RATIOS**

Solutions were prepared following the description in the experimental section. Labelling follows a convention frequently used in the description of ternary blends. With densities for PC_70_BM of 1.62 g/cm^3^, Cy7T of 1.519 g/cm^3^ and PBDTTT-C of 1.5 g/cm^3^, we arrive at the following volume fractions:

**Table S1.** **Summary and composition of all samples.**

| PC_70_BM: Cy7T: PBDTTT-C | Volume fraction | | |
| --- | --- | --- | --- |
| Label | PC_70_BM | Cy7T | PBDTTT-C |
| 1.5: 0.0: 1.0 | 58.1 | 0 | 41.9 |
| 1.5: 0.1: 1.0 | 55.8 | 4 | 40.2 |
| 1.5: 0.3: 1.0 | 51.7 | 11 | 37.2 |
| 1.5: 0.5: 1.0 | 48.2 | 17.1 | 34.7 |
| A (0.0: 3.0: 1.0) | 0 | 75 | 25 |
| B (0.0: 1.0: 1.0) | 0 | 50 | 50 |
| C (0.0: 0.3: 1.0) | 0 | 25 | 75 |
| D (1.5: 4.2: 0.0) | 25 | 75 | 0 |
| E (1.5: 1.4: 0.0) | 50 | 50 | 0 |
| F (1.5: 0.47: 0.0) | 75 | 25 | 0 |
| G (2.2: 1.0: 1.0) | 50 | 25 | 25 |
| H (0.54: 0.51: 1.0) | 25 | 25 | 50 |
| I (0.36: 0.0: 1.0) | 25 | 0 | 75 |
| K (3.2: 0.0; 1.0) | 75 | 0 | 25 |
| L (1.1: 1.0: 1.0) | 33 | 33 | 33 |
| M (19.4: 1.0: 1.0) | 90 | 5 | 5 |
| N (1.1; 2.0: 1.0) | 25 | 50 | 25 |

**ABSORPTION SPECTRA OF TERNARY BLEND FILMS**

Figure S1. Normalized attenuance spectra of PC_70_BM: Cy7T: PBDTTT-C and PC_70_BM: Cy7P: PBDTTT-C blend films with different contents of Cy7. In the blends containing Cy7P, the main absorption peaks are present, but scattering induced by the morphology influences the peak intensity.

**EFFCT OF FILM THICKNESS ON EQE**

It is demonstrated that the shape of the EQE can be stronlgy influenced by the film thickness. While the absorbance spectra scale with thickness, in the EQE positions and intensities of peaks are changing. The thinner the films, the more does the EQE follow film absorbance. Deviations in thicker films can be due to filter effects, but also due to decreased photoconductivity in deeper layers of the film. At maximum absorption not the entire film is illuminated, leading to a loss in photoconductivity and consequentially poor charge extraction. The EQE is actually largest where the absorbance has a minimum, namely at 510 nm. Particularly, while the main absorption peak of PC_70_BM is at λ = 450 nm, the PC_70_BM EQE main peak wanders from λ = 550 nm in 140 nm thick films to λ = 430 nm in thinner films. We do not have a straightforward explanation for the second EQE peak at λ = 400 nm for the thickest film.

Figure S2. Absorbance and EQE spectra of binary blend films of different thickness of PC_70_BM: PBDTTT-C with weight ratios of 1.5: 1.0.

**SOLAR CELL PARAMETERS EXTRACTED FROM J-V-CURVES (REVERSE SATURATION CURRENT AND IDEALITY FACTOR)**


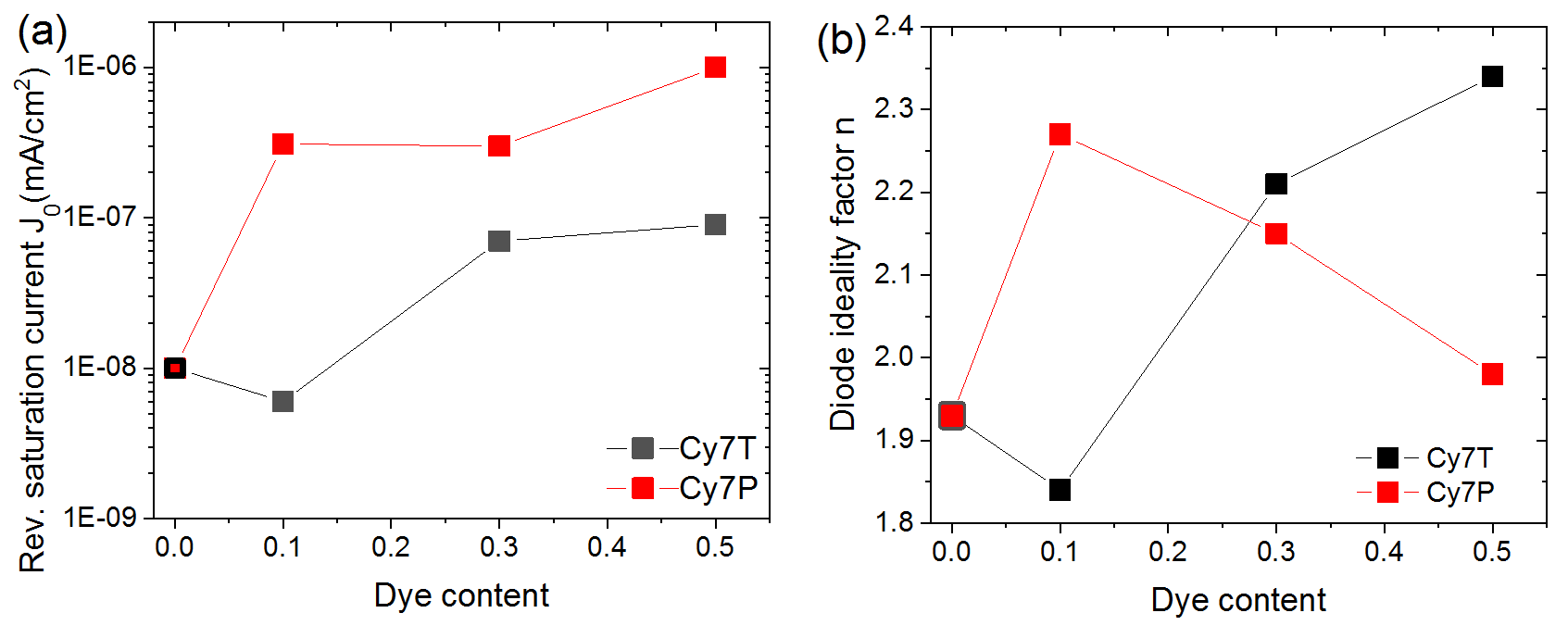


Figure S3. Solar cell parameters extracted from the J-V curves. (a) Reverse saturation current J_0_. (b) Diode ideality factor n.

**DETERMINATION OF PHASE DIAGRAM FROM SELECTIVE DISSOLUTION**

The procedure for the construction of a ternary phase diagram by selectively dissolving an individual phase is exemplified with the binary blends Cy7T: PBDTTT-C and PC_70_BM: Cy7T, and one ternary blend composition. For the Cy7T: PBDTTT-C blend, the dye is completely removed from the film when immersed in ACN (Figure S4(a)). Here dye and polymer separate in nearly pure phases. In the PC_70_BM: Cy7-T system, the dye can never be fully dissolved from the film (Figure S4(b)). We can conclude that a stable α-phase exists that contains significant amounts of Cy7T. Conversely, we can say that significant amounts of Cy7T are soluble in the fullerene. Similarly, in the ternary blend we can find a composition region where ACN does not influence the film absorption at all (Figure S4(c)). This is a strong sign for the existence of a single-phase. The analysis is systematically performed for a number of compositions across the phase space. UV-vis spectra are shown in the remaining tablets of Figure S4. From the data we can sketch a rough course of the single-, two- and three-phase regions of the ternary phase diagram (Figure 5(c)).

**
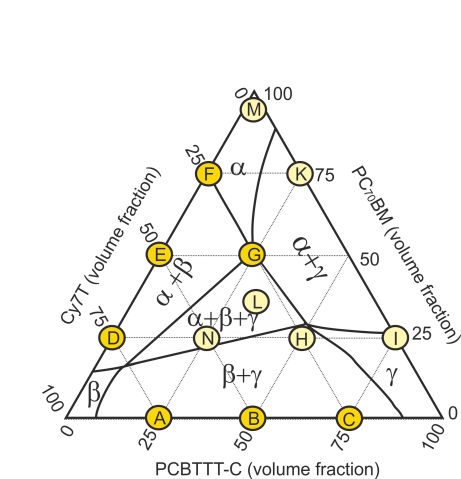

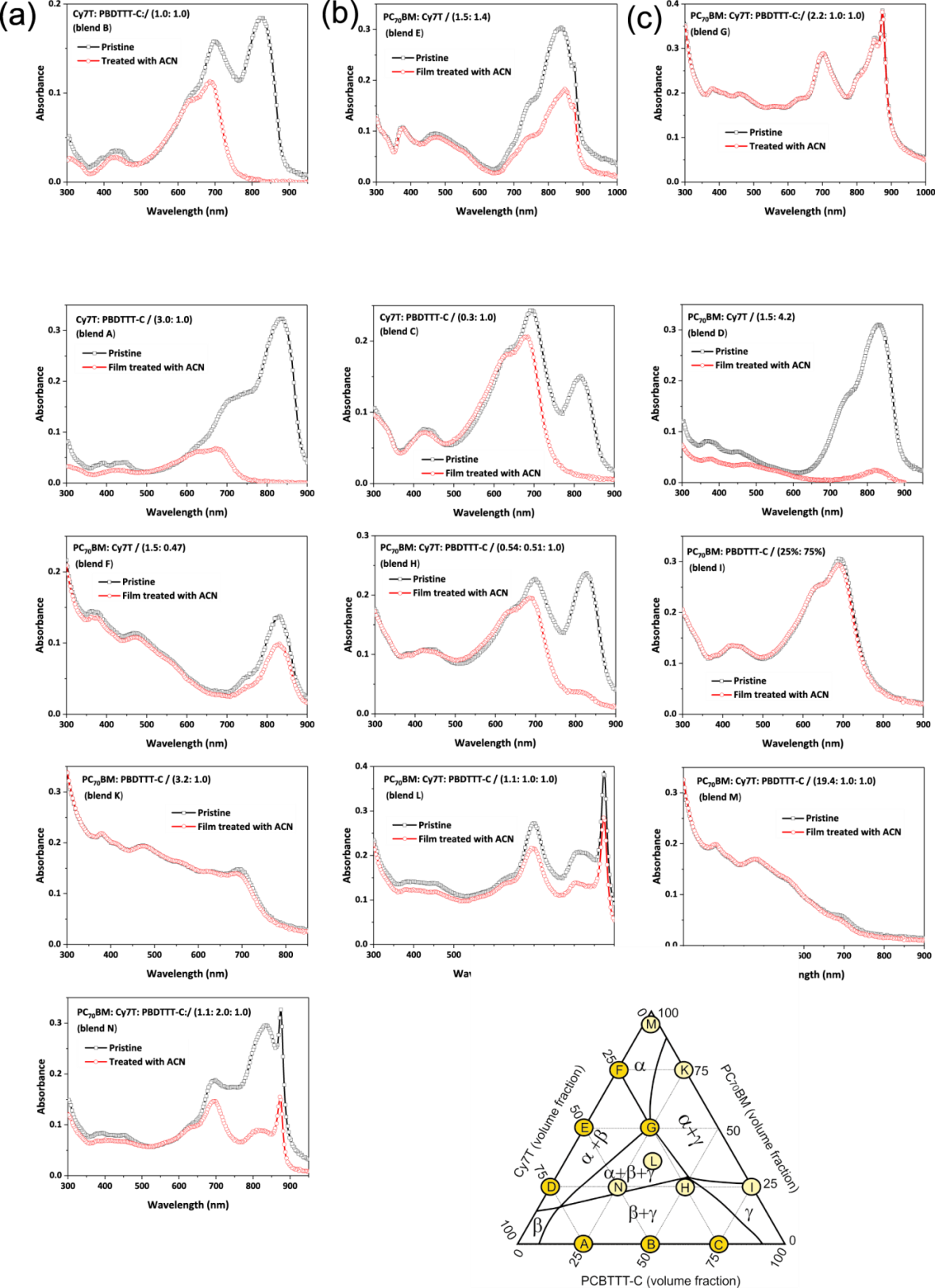
**

Figure S4. UV-Vis spectra of blend films of PC_70_BM: Cy7T: PBDTTT-C with different blend compositions before and after immersion in ACN. The resulting phase diagram is sketched at the bottom, including labels of the probed samples.

**NANO IR IMAGES**

Figure S5. 2 μm × 2 μm AFM morphology images of PC_70_BM: Cy7T: PBDTTT-C films. IR spectra are collected at four different spots of the sample (marked on the AFM image on the left) using a QCL source. IR bands characteristic for PC_70_BM are visible (1428 cm^-1^, 1452 cm^-1^ and 1738 cm^-1^. The broad band at 1608 cm^-1^ belongs to the polymer (conjugated chain).


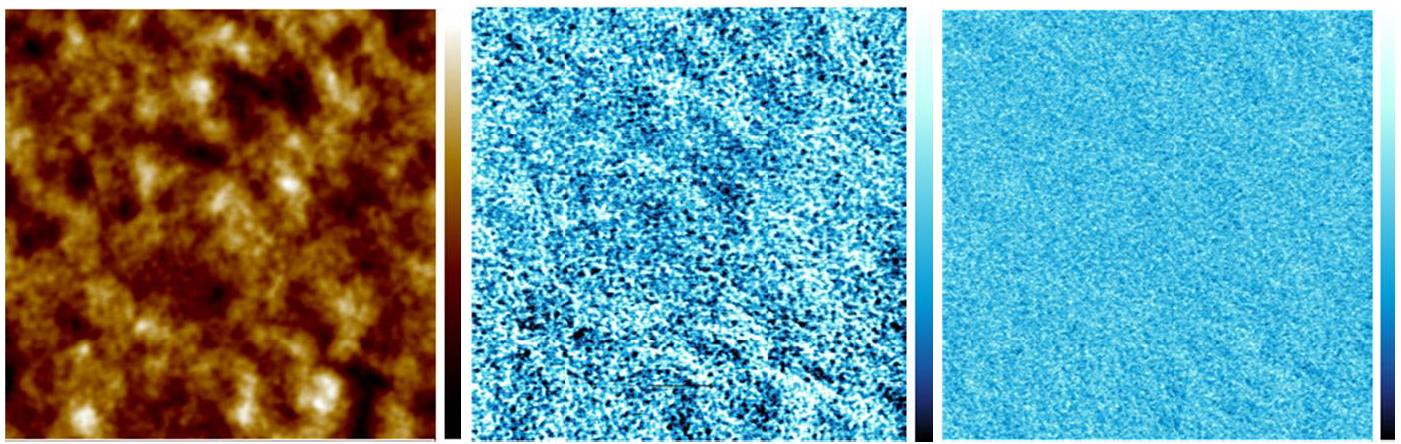


(c)

(b)

(a)

Figure S6. (a, b) same images as Figure 5 in the main text: Tapping AFM-IR image of a ternary blend of PC_70_BM: Cy7T: PBDTTT-C (Cy7T05). (a) Topography (b) IR image collected at 1736 cm^-1^ (PC70BM). (c) IR image collected at 1800 cm^-1^ (no absorption). Image size: 5 μm × 5 μm. The data scale of the height images is 50 nm, the data scale of the IR signal is 0.50 mV.

**SATURATION CURRENT**

Additional information can be extracted from the J-V-curves when plotting J_ph_ = J_L_-J_D_, where J_L_ and J_D_ are the photocurrent densities under AM 1.5G illumination and in the dark, respectively [1] versus an effective voltage. The effective voltage V_eff_ is defined as: V_eff_=V_0_-V, where V is the applied voltage and V_0_ is the voltage at which J_ph_= 0. The ratio of J_ph_ and J_sat_ can be interpreted as product of charge generation and collection efficiency [2].

Figure S7 presents the photocurrent density J_ph_/J_sat_ versus effective voltage for the different devices. J_sat_ was defined as J_ph_ at V_eff_ = 1.0 V. All ternary blends showed a similar behavior, differing from the binary blend. For none of the samples saturation was reached at V_eff_ = 1.0 V.

Figure S7. Photocurrent density J_ph_/ J_sat_ versus effective voltage V_eff_ curves for the binary and ternary blend OSCs under constant incident light intensity (AM 1.5G, 100 mW cm^-2^).

**CHARGE CARRIER MOBILITY**

Hole only and electron only devices were fabricated using the following architectures: ITO/ PEDOT:PSS/ PC_70_BM: Cy7T: PBDTTT-C/ MoO_3_/ Ag and Al/ PC_70_BM: Cy7T: PBDTTT-C/ Al for holes and electrons, respectively. The carrier mobility was calculated by the space-charge limited current (SCLC) model from the Mott–Gurney equation based on the J–V curves shown in Figure S8, assuming a field independent mobility. J is the current density, ε_0_ is the vacuum permittivity, ε_r_ is the dielectric constant (ε_r_ := 3 for common organic materials), μ is the charge carrier mobility, V is the applied voltage, and L is the active layer thickness [3, 4].

$J=\frac{9}{8}\varepsilon_{0}\varepsilon_{r}\mu\frac{V^{2}}{L^{3}}$ (1)

Figure S8. J-V2 characteristics of (a) hole only device: ITO/ PEDOT:PSS/ PC_70_BM: Cy7T: PBDTTT-C/ MoO3/ Ag and (b) electron only device: Al/ PBDTTT-C: PC_70_BM: Cy7T/ Al with different Cy7T doping ratios.

**EQE IN THE REMAINING TWO-PHASE REGIONS (α + β and β + γ) FOR BINARY BLENDS, THREE-PHASE REGION (α + β + γ) AND THE α-PHASE**

The EQE of the binary blends PC_70_BM: Cy7T (Figure S9(b)) showed only a significant response from the dye absorption region. Dye aggregation here becomes a major factor. The absorption region of the dye expanded from 700 nm (H-aggregate formation) to 900 nm (J-aggregate formation) and also photocurrent is generated from these regions. We have reported and discussed dye aggregates in blend films with PC_60_BM [5, 6], and will not address this topic in the context of this paper. In ongoing work we showed a J-aggregate arrangement of Cy7T in single crystals. We furthermore refer to a number of publications where dye aggregation is discussed in the context of artificial light harvesting [7, 8]. Still, the EQE response can be understood from our earlier elaborations: a BHJ type morphology is present at a length scale in the range of a couple of 10 nms. Following our model, the PCBM-rich phase (α-phase) contains significant amounts of dye. According to the energy levels (Figure 1), excitons generated in the dye can dissociate at the PCBM interface and charges can be extracted. Differently, contribution from PC_70_BM to the EQE is weak, excitons will be quenched by the dye intermixed in the α -phase.

The EQE values in the Cy7T: PBDTTT-C blend (Figure S9(c)) were generally low. This is anticipated from the similar HOMO and LUMO levels.

The ternary blend system with composition (1.1: 1.0: 1.0) is believed to fall into the three-phase region (Figure S9(d)). Accordingly, the shape of the EQE (Figure S9(e)) underwent a marked transition and did not show any concordance with the spectra from the two-phase (α + γ) region (Figure 2(b)), rather, in the dye absorption region, the EQE spectrum resembled the spectrum from the (α + β) two-phase region. A direct contribution from PBDTTT-C was absent, the EQE peak from PC_70_BM may result from a (α + γ) phase structure. We understand this observation as a very strong confirmation for our model which claims that all samples Cy7T01 – Cy7T05 fall into the two-phase region.

We also probed the EQE in the single-phase region (Figure S9(f)). A small photocurrent was generated from PC_70_BM, EQE peak maximum here was lying at 400 nm, and the dye also contributed to charge generation.


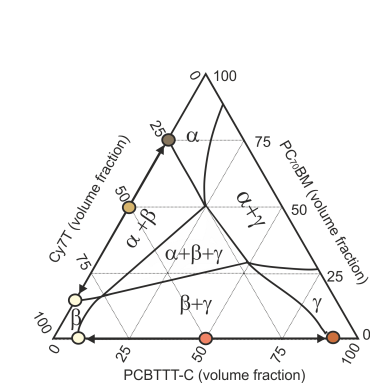

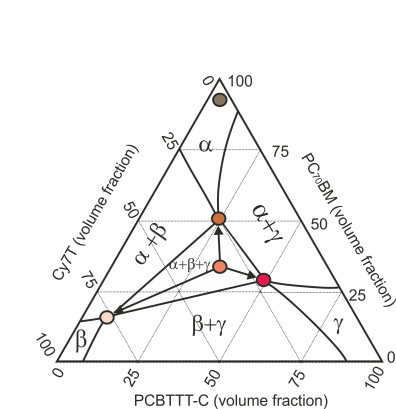

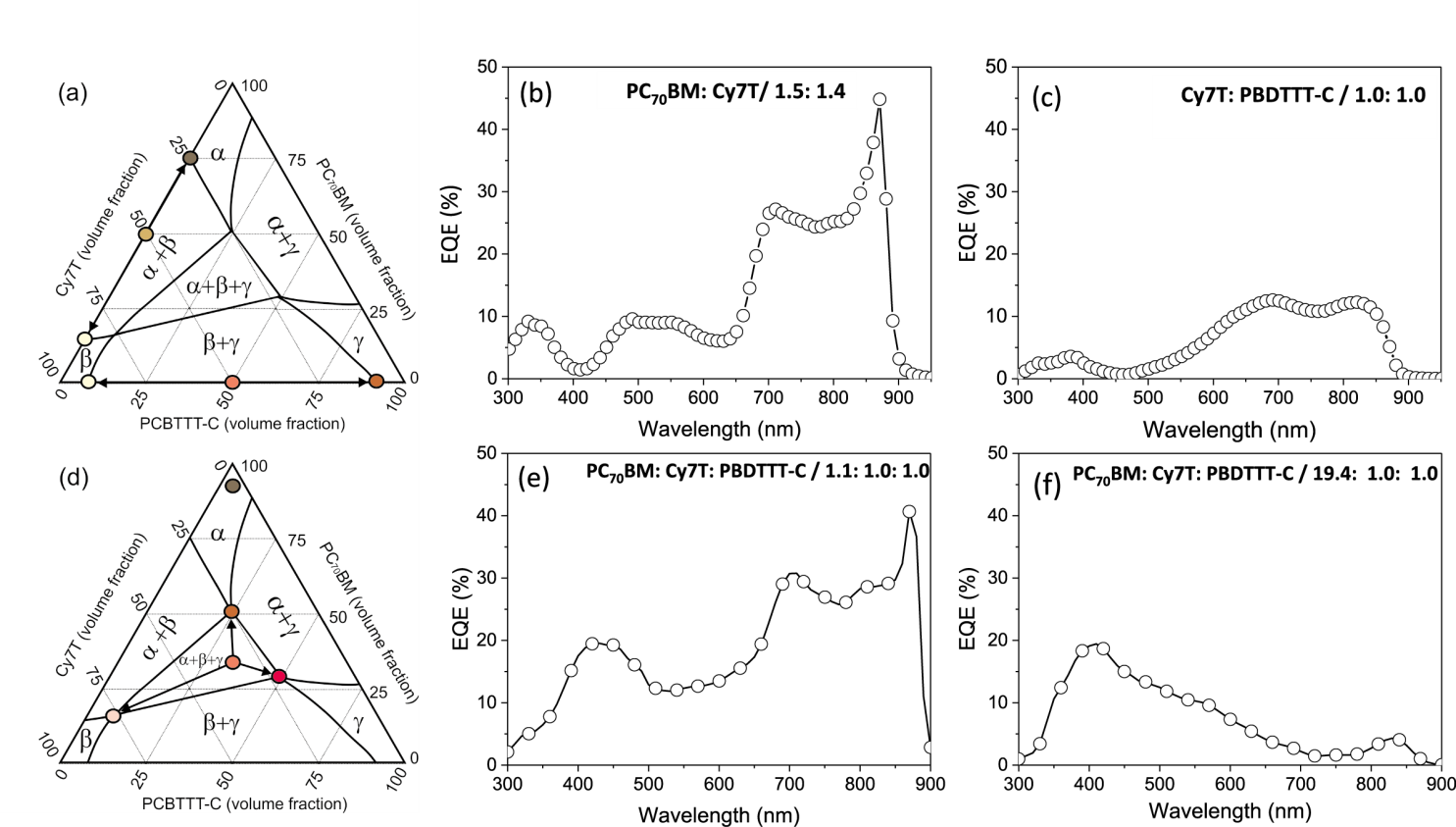


Figure S9. (a) Schematics of initial and final composition of the binary blend samples shown in (b) and (c). The binary blends separate into two phases. EQE spectra of the binary blends (b) PC70BM: Cy7T and (c) Cy7T: PBDTTT-C. (d) Schematics of initial and final composition of a ternary blend sample separating into three phases. The EQE spectrum is shown in (e). (f) EQE of a sample from the one phase region. The composition is indicated in (d).

**REFERENCES**

1. Ooi ZE, Jin R, Huang J, et al. On the pseudo-symmetric current-voltage response of bulk heterojunction solar cells. Journal of Materials Chemistry. 2008;18(14):1644-1651. doi: 10.1039/b718563d.

2. Faist MA, Shoaee S, Tuladhar S, et al. Understanding the Reduced Efficiencies of Organic Solar Cells Employing Fullerene Multiadducts as Acceptors. Advanced Energy Materials. 2013;3(6):744-752. doi: 10.1002/aenm.201200673.

3. Zhang M, Zhang FJ, An QS, et al. High efficient ternary polymer solar cells based on absorption complementary materials as electron donor. Solar Energy Materials and Solar Cells. 2015;141:154-161. doi: 10.1016/j.solmat.2015.05.037.

4. Foster S, Deledalle F, Mitani A, et al. Electron Collection as a Limit to Polymer:PCBM Solar Cell Efficiency: Effect of Blend Microstructure on Carrier Mobility and Device Performance in PTB7:PCBM. Advanced Energy Materials. 2014;4(14). doi: 10.1002/aenm.201400311.

5. Heier J, Steiger R, Nuesch F, et al. Fast Assembly of Cyanine Dyes into Aggregates onto 6,6 -Phenyl C-61-Butyric Acid Methyl Ester Surfaces from Organic Solvents. Langmuir. 2010;26(6):3955-3961. doi: 10.1021/la903313k.

6. Heier J, Steiger R, Hany R, et al. Template synthesis of cyanine dye H-aggregates on nanostructured 6,6 -phenyl C-61-butyric acid methyl ester substrates. Physical Chemistry Chemical Physics. 2011;13(34):15714-15722. doi: 10.1039/c1cp21485c.

7. Scholes GD. Designing light-harvesting antenna systems based on superradiant molecular aggregates. Chemical Physics. 2002;275(1-3):373-386. doi: 10.1016/s0301-0104(01)00533-x.

8. Olaya-Castro A, Scholes GD. Energy transfer from Forster-Dexter theory to quantum coherent light-harvesting. International Reviews in Physical Chemistry. 2011;30(1):49-77. doi: 10.1080/0144235x.2010.537060.
